# Interação entre Periódicos da Ciência Cardiovascular no Brasil: Um Formato que Deve Ser Melhor Explorado

**DOI:** 10.36660/abc.20200159

**Published:** 2020-03-20

**Authors:** Carlos Eduardo Rochitte

**Affiliations:** 1 Universidade de São PauloFaculdade de MedicinaHospital das ClínicasSão PauloSPBrasilUniversidade de São Paulo - Faculdade de Medicina Hospital das Clínicas - Instituto do Coração, São Paulo, SP – Brasil

**Keywords:** Bibliometria, Fator de Impacto de Revistas, Publicações Periódicas como Assunto/tendências, Base de Dados

No ambiente de grande competição entre periódicos científicos, em que a batalha pelo melhor artigo científico, maior número de citações e maior fator de impacto (FI) parece não ter limites, os Arquivos Brasileiros de Cardiologia (ABC Cardiol) tem tomado ações no intuito de contribuir para o crescimento de outros periódicos com foco similar na comunidade científica nacional. O objetivo principal é permitir que a nossa melhor ciência seja veiculada por periódicos científicos de qualidade cada vez mais alta, obtenha a visibilidade que merece, e possa contribuir de forma mais impactante na comunidade científica mundial.

Com o atual FI próximo a 1,7 pela *Journal of Citation Reports* (JCR), os ABC Cardiol têm tido um aumento crescente de submissões nacionais e principalmente internacionais.^1,2^Nossa taxa de aceitação não passa de 15% e com tendência a diminuir ainda mais. Nesse cenário, muitos artigos de qualidade significativa não podem ser aceitos para publicação nos ABC Cardiol, incluindo artigos oriundos dos nossos profícuos programas de pós-graduação. Infelizmente, tal fato tem como efeito indesejável a diminuição da exposição da nossa melhor ciência em fontes de indexação como o PubMEd, Scielo e outros.

Com essa situação em mente, o corpo editorial dos ABC Cardiol tem comunicado aos autores desses artigos não aceitos para publicação a possibilidade de indicação ou transferência da submissão desses manuscritos para periódicos nacionais em ascensão, que poderiam ser adequados para a divulgação daquela ciência específica. É importante ressaltar que a decisão de transferência ou submissão a periódicos sugeridos é uma decisão única e exclusiva dos autores. Essa é uma logística do tipo “win-win situation”, pois ganham tanto os autores que podem ter seus artigos publicados de forma ágil em periódicos de prestígio e em crescimento, como os periódicos que terão probabilidade de maior número de citação e poderão melhorar e ampliar sua indexação em bases científicas mundiais.

Como exemplos de colaboração, temos a mais íntima entre os ABC Cardiol e o *International Journal of Cardiovascular Sciences* (IJCS). Ambas são mantidas pela SBC e indexadas na Scielo, o que permite que ambas utilizem o mesmo sistema de submissão, o ScholarOne, e possam realizar transferências diretas, com as revisões incluídas, desde que autorizadas pelos autores. Os ABC Cardiol têm um foco mais intenso em doença cardiovascular, e o IJCS na multidisciplinaridade, incluindo aspectos da nutrição, fisioterapia entre outros, o que torna a relação entre esses periódicos sinérgica e não competitiva. Esta parceria com o editor-chefe do IJCS, Dr. Claudio Tinoco Mesquita, tem impulsionado ambos os periódicos, de forma muito efetiva, à maior visibilidade e impacto.

Em modo de colaboração intermediária, a ABC Imagem Cardiovascular e a *Journal of Transcatheter Interventions *(JOTCI) formam, com os ABC Cardiol, uma grande família de periódicos científicos na área da ciência cardiovascular. Contudo, esses periódicos não fazem parte da Scielo, não permitindo, assim, sua integração ao sistema do ScholarOne sem custo associado. Por isso, quando um artigo é rejeitado para publicação pelos ABC Cardiol, o modelo inclui o envio de e-mail aos autores, contendo um *link* para o contato direto com os periódicos que, na visão do corpo editorial dos ABC Cardiol, seriam adequados para veiculação daquele material científico. Isso facilita e agiliza uma possível submissão pelos autores.

Neste ponto, gostaria de fazer um agradecimento pessoal ao editor-chefe da JOTCI, Dr. Pedro Beraldo de Andrade, pela divulgação dessa colaboração em um editorial publicado em sua revista, que comentou também sobre a primeira publicação internacional gerada por esse modelo na JOTCI.^3^Também agradeço ao editor-chefe Dr. Silvio Henrique Barberato da ABC Imagem Cardiovascular que tem mantido uma parceria muito próxima com os ABC Cardiol.

Ainda, julgamos importante implementar uma colaboração com outras sociedades de especialidade mantenedoras de periódicos de grande importância para nossa ciência nacional, como o Jornal Brasileiro de Pneumologia e o *Brazilian Journal of Cardiovascular Surgery*. Fica aqui a ideia para discutirmos com os respectivos editores chefes Dr. Bruno Guedes Baldi e Dr. Domingos M. Braile.

A proximidade dos periódicos em modelo de família permite maior sucesso de cada um dos periódicos individualmente. Essa tem sido a rotina na ciência mundial, em especial na área de cardiologia, a exemplo do crescimento impressionante da família de periódicos do *Journal of the America College of Cardiology* (JACC), ligada ao *American College of Cardiology *dos Estados Unidos, e da família de periódicos da *European Heart Journal* (EHJ), ligada a *European Society of Cardiology *(ESC) na Europa. Apenas como exemplo, entre 2011 e 2018, sete curtos anos em tempo editorial, o EHJ saiu de um FI de 10,4 para 24,8, o *European Journal of Heart Failure* de 4,8 para 13,9, o *European Journal of Preventive Cardiology* de 2,6 para 5,6, *EHJ Cardiovascular Imaging* de 2,3 para 5,2, e o *Europace* de 1,9 para 6,1. Esses números são realmente impressionantes em termos de aumento de FI em um período muito curto. Este modelo parece ser muito eficaz em melhorar a divulgação da ciência e permitir ao mesmo tempo o crescimento do FI dos periódicos membros da família.

Como família ou como “amigos”, creio que a colaboração efetiva entre periódicos nacionais de grande reputação permite um crescimento do grupo de periódicos que não seria possível isoladamente. Espero que essa colaboração inicial e preliminar possa estimular uma maior discussão e interação futura mais intensa entre periódicos com objetivos comuns e focos científicos próximos. Convido a todos a pensarmos juntos em modelos que permitam real crescimento da nossa comunidade científica dentro da ciência mundial. A qualidade da nossa produção científica já é fato; precisamos minimizar as dificuldades que ainda temos de divulgar efetivamente nossa melhor ciência. Este deve ser um trabalho conjunto dos nossos periódicos científicos nacionais.

Entre as ações do ABC Cardiol nesta direção estão a modernização do portal digital do periódico e utilização de ferramentas digitais modernas. Entre elas ferramentas que sugiram artigos semelhantes em outros periódicos quando uma busca é realizada em seu portal digital, com foco nos periódicos nacionais da família cardiovascular. As oportunidades são muitas e temos que trabalhar para aproveitá-las integralmente.
