# Supplementary material for: Interação entre Periódicos da Ciência Cardiovascular no Brasil: Um Formato que Deve Ser Melhor Explorado
Source: Arq Bras Cardiol. 2020 Mar 20;114(3):433–4. [Article in Portuguese] doi: 10.36660/abc.20200159 (PMC7792741; doi:10.36660/abc.20200159)
Supplement: Translation

# Translation: Interaction among Cardiovascular Scientific Journals in Brazil: A Model that should be Better Explored

**Authors:** Carlos Eduardo Rochitte

**Affiliations:** 1 BrazilUniversidade de São Paulo - Faculdade de Medicina Hospital das Clínicas - Instituto do Coração, São Paulo, SP – Brazil

**Keywords:** Bibliometrics, Journal Impact Factor, Periodicals as Topic/trends, Database

In the environment of intense, and apparently unlimited competition among scientific journals for the best article, and the highest number of citations and impact factor (IF), the *Arquivos Brasileiros de Cardiologia* (ABC Cardiol) has taken actions to foster the growth of other journals with similar scope in the national scientific community. The main objective is to promote dissemination of our best science by journals of increasingly higher quality, so that it can attract deserving attention and make a stronger contribution to the world scientific community.

With the current IF of nearly 1.7 according to the Journal of Citation Reports (JCR), the ABC Cardiol has attracted more and more submissions from national and international authors.^1,2^ Our acceptance rate is no more than 15% and tends to decrease even more. In this scenario, many papers of significant quality cannot be accepted for publication in the ABC Cardiol, including articles from studies developed in our successful postgraduate programs. This has an undesirable effect – the reduced exposure of our best science in indexation sources such as PubMED, Scielo, among others.

With this in mind, the editorial board of the ABC Cardiol has suggested to the authors of manuscripts not accepted for publication, either the possibility of recommendation of these papers for publication, or the transfer of these papers to other potential national journals that would be adequate for dissemination of that specific scientific information. It is important to point out that the decision to transfer or submit the manuscript to one of the journals recommended by the ABC Cardiol is solely and exclusively made by the authors. This is a “win-win situation” where both authors and journals win, the first for having their manuscript rapidly published by high quality journals, and the second for increasing the likelihood of citations and indexation in international scientific databases.

One example of such collaboration is the up-close relationship between the ABC Cardiol and the International Journal of Cardiovascular Sciences (IJCS), both held by the SBC and indexed in Scielo. This allows that articles are submitted through the same unique system, the ScholarOne, and can be directly transferred, including the revisions, once authorized by the authors. While the ABC Cardiol focuses more on cardiovascular disease, the IJCS focuses more on multidisciplinarity, including other areas such as Nutrition and Physiotherapy, which makes the relationship between these journals synergistic rather than competitive. Thus, the partnership with the Editor-in-Chief of the IJCS, Dr. Claudio Tinoco Mesquita, has effectively boosted the recognition and the impact of both journals.

In an intermediate level of collaboration, the *ABC Imagem Cardiovascular* and the Journal of Transcatheter Interventions (JOTCI), together with the ABC Cardiol, constitute a large family of scientific journals in the cardiovascular field. However, these journals are not members of Scielo, and hence cannot be integrated to the ScholarOne system without additional cost. For this reason, when a manuscript is rejected for publication by the ABC Cardio, an e-mail is sent to the authors, containing a link to journals that, according to the ABC Cardiol’s editorial board, may be adequate to publish that scientific material. This model facilitates and speeds up the (re)submission of the manuscript.

At this point, I would like to thank the Editor-in-Chief of the JOTCI, Dr. Pedro Beraldo de Andrade, for reporting about this collaboration in an editorial published in the JOTCI, and commenting about the first publication of an article resulting from this model in an international journal.^3^ I would also like to thank the Editor-in-Chief Dr. Silvio Henrique Barberato of the *ABC Imagem Cardiovascular* for the close relationship with the ABC Cardiol.

Also, we find it important to implement a collaboration to other societies that support other journals relevant to the national science, such as the* Jornal Brasileiro de Pneumologia* and the Brazilian Journal of Cardiovascular Surgery. This is my suggestion for consideration by the respective Editors-in-Chief Dr. Bruno Guedes Baldi and Dr. Domingos M. Braile.

The proximity of the journals in a “family” model allows a greater success of each journal individually. This has been a tendency of the world science, particularly in Cardiology, as exampled by the impressive growth of the American College of Cardiology (JACC)’s family of journals, associated with the USA American of Cardiology, and the European Heart Journal’s (EHJ) family, associated with the European Society of Cardiology (ESC) in Europe. As example, in the period from 2011 and 2018 (short seven editorial years), the IF of the EHJ increased from 10.4 to 24.8, the European Journal of Heart Failure had its IF increase from 4.8 to 13.9, the European Journal of Preventive Cardiology from 2.6 to 5.6, the EHJ Cardiovascular Imaging from 2.3 to 5.2, and the Europace from 1.9 to 6.1. These increases in IF, in such a short period of time, are impressive. Therefore, this model seems very effective in improving the dissemination of science while enabling the increase of the IF of each member of the family.

Either as a family or as “friends”, I believe that an effective collaboration between national renowned journals allows the growth of a group of journals that would not be seen by the journals alone. Hopefully, this initial and preliminary collaboration will lead to a broader discussion and closer interaction between journals with similar aims and scope. I invite everyone to think together about models that would increase the visibility of our scientific papers in the world science. Although the high quality of our scientific production is already recognized, we still need to minimize the barriers to effectively disseminate our science. This must be a joint effort of the Brazilian scientific journals.

Among the actions of ABC Cardiol in this direction are the reshaping of the journal’s digital portal and the use of modern digital tools. Among them are tools that suggest similar articles in other journals when a search is carried out on its digital portal, focusing on national journals in the cardiovascular family. The opportunities are many and we have to work to take full advantage of them.
